# Label-Free Quantitative Proteomics of Lysine Acetylome Identifies Substrates of Gcn5 in Magnaporthe oryzae Autophagy and Epigenetic Regulation

**DOI:** 10.1128/mSystems.00270-18

**Published:** 2018-11-20

**Authors:** Meiling Liang, Shulin Zhang, Lihong Dong, Yanjun Kou, Chaoxiang Lin, Weijun Dai, Lian-Hui Zhang, Yi Zhen Deng

**Affiliations:** a State Key Laboratory for Conservation and Utilization of Subtropical Agro-Bioresources, South China Agricultural University, Guangzhou, China; b Integrative Microbiology Research Centre, Guangdong Province Key Laboratory of Microbial Signals and Disease Control, South China Agricultural University, Guangzhou, China; c State Key Laboratory of Rice Biology, China National Rice Research Institute, Hangzhou, China; Princeton University

**Keywords:** comparative acetylome, Gcn5, histone acetyltransferase (HAT), *Magnaporthe oryzae*

## Abstract

The rice blast fungus Magnaporthe oryzae poses a great threat to global food security. During its conidiation (asexual spore formation) and appressorium (infecting structure) formation, autophagy is induced, serving glycogen breakdown or programmed cell death function, both essential for *M. oryzae* pathogenicity. Recently, we identified an M. oryzae histone acetyltransferase (HAT) Gcn5 as a key regulator in phototropic induction of autophagy and asexual spore formation while serving a cellular function other than autophagy induction during *M. oryzae* infection. To further understand the regulatory mechanism of Gcn5 on *M. oryzae* pathogenicity, we set out to identify more Gcn5 substrates by comparative acetylome between the wild-type (WT) and *GCN5* overexpression (OX) mutant and between OX mutant and *GCN*5 deletion (knockout [KO]) mutant. Our results showed that Gcn5 regulates autophagy induction and other important aspects of fungal pathogenicity, including energy metabolism, stress response, cell toxicity and death, likely via both epigenetic regulation (histone acetylation) and posttranslational modification (nonhistone protein acetylation).

**IMPORTANCE** Gcn5 is a histone acetyltransferase that was previously shown to regulate phototropic and starvation-induced autophagy in the rice blast fungus Magnaporthe oryzae, likely via modification on autophagy protein Atg7. In this study, we identified more potential substrates of Gcn5-mediated acetylation by quantitative and comparative acetylome analyses. By epifluorescence microscopy and biochemistry experiments, we verified that Gcn5 may regulate autophagy induction at both the epigenetic and posttranslational levels and regulate autophagic degradation of a critical metabolic enzyme pyruvate kinase (Pk) likely via acetylation. Overall, our findings reveal comprehensive posttranslational modification executed by Gcn5, in response to various external stimuli, to synergistically promote cellular differentiation in a fungal pathogen.

## Abstract (precis)

Gcn5 is a histone acetyltransferase that was previously shown to regulate phototropic and starvation-induced autophagy in the rice blast fungus Magnaporthe oryzae, likely via modification on autophagy protein Atg7. In this study, we identified more potential substrates of Gcn5-mediated acetylation by quantitative and comparative acetylome analyses. By epifluorescence microscopy and biochemistry experiments, we verified that Gcn5 may regulate autophagy induction at both the epigenetic and posttranslational levels and regulate autophagic degradation of a critical metabolic enzyme pyruvate kinase (Pk) likely via acetylation. Overall, our findings reveal comprehensive posttranslational modification executed by Gcn5, in response to various external stimuli, to synergistically promote cellular differentiation in a fungal pathogen.

## INTRODUCTION

Histone acetyltransferases (HATs) and histone deacetylation complexes (HDACs) are known to catalyze acetylation/deacetylation of histones on specific lysine residues and thus, epigenetically regulate global gene transcription, stress reaction, and metabolic flux in plants and yeasts (1–3). In addition, evidence is accumulating that HATs and HDACs are also able to directly acetylate or deacetylate cytosolic proteins besides histone proteins (4–6). Recent studies revealed that fungal HATs are involved in fungal virulence and pathogenic morphogenesis (7–14). However, our knowledge on the underlying mechanism, including the HAT or HDAC substrates and the relevant cellular events, is still limited (15, 16).

Magnaporthe oryzae is an important fungal pathogen that causes the devastating blast disease in rice and several other crops (17). M. oryzae initiates its pathogenic life cycle by forming asexual spores, conidia, upon exposure to light (18). Our recent studies showed that carbohydrate catabolism and homeostasis are spatially and temporally regulated via autophagy to ensure successful conidiation in *M. oryzae* (19, 20). Phototropic regulation of autophagy was recently found to be mediated by Gcn5, a histone acetyltransferase that could acetylate autophagy protein Atg7 to repress autophagy, in *M. oryzae* (21). Gcn5 was also shown to be an important regulator of *M. oryzae* pathogenicity (21), but the exact substrates, other than Atg7, of Gcn5-catalyzed posttranslational modifications (PTM) that are relevant to fungal pathogenicity, are not fully known. Autophagy was also demonstrated as essential for *M. oryzae* infection, likely by serving a function in programmed cell death during appressorium maturation (22) or in maintaining lipid body integrity (23).

Here, we present the identification of potential target proteins of *M. oryzae* Gcn5 via quantitative acetylome analysis. We noticed that carbon metabolism, oxidative phosphorylation, and cell death are mediated directly or indirectly by Gcn5-catalyzed acetylation on nonhistone, cytosolic proteins. Furthermore, our experimental results revealed that Gcn5 regulated autophagy induction by a combination of posttranslational modifications and epigenetic regulation of gene expression and regulated autophagic degradation of pyruvate kinase (Pk) likely via acetylation of Pk protein. These findings provide an integrative regulation of morphogenesis and/or infection-related autophagy and its potential substrate(s) for degradation for a fungal pathogen.

## RESULTS AND DISCUSSION

### Identification of phototropic induced lysine acetylation sites.

To identify more Gcn5 substrates and investigate their function in *M. oryzae* pathogenicity, we developed a robust workflow for quantitative acetylome as depicted in Fig. S1 in the supplemental material. Mycelia from *GCN5OX* (overexpression [OX]) and wild-type (WT) strains were exposed to light for 12 h, to maximize phototropic induction of protein acetylation, before total protein lysis. To control for process variability, three independent sets of mycelial samples per strain were prepared in parallel for MS analysis. Each sample was digested with trypsin, and peptides containing AcK were immunoprecipitated using PTMScan Acetyl-Lysine Motif (Ac-K) kit (Cell Signaling Technology, catalog no. 13416S). The enriched AcK peptides were analyzed in triplicate by LC-MS/MS on a Q Exactive mass spectrometer (Thermo Fisher Scientific, US), and data were searched against the *M. oryzae* proteome.

10.1128/mSystems.00270-18.1 FIG S1 Experimental flow of comparative acetylome analysis with WT and OX strains. Mycelia were harvested from WT or OX colonies grown on 10 PA solid medium plates for total protein lysis and trypsin digestion. Three independent biological replications were sampled for each strain. The acK peptides were immunoprecipitated using PTMScan Acetyl-Lysine Motif (Ac-K) kit (Cell Signaling Technology, catalog no. 13416S). Enriched peptides were separated and analyzed in duplicate by LC-MS/MS, followed by bioinformatic analyses to identify differentially acetylated proteins between WT and OX strain. Download FIG S1, TIF file, 0.8 MB. Copyright © 2018 Liang et al. 2018 Liang et al. This content is distributed under the terms of the Creative Commons Attribution 4.0 International license.

**FIG 1:**
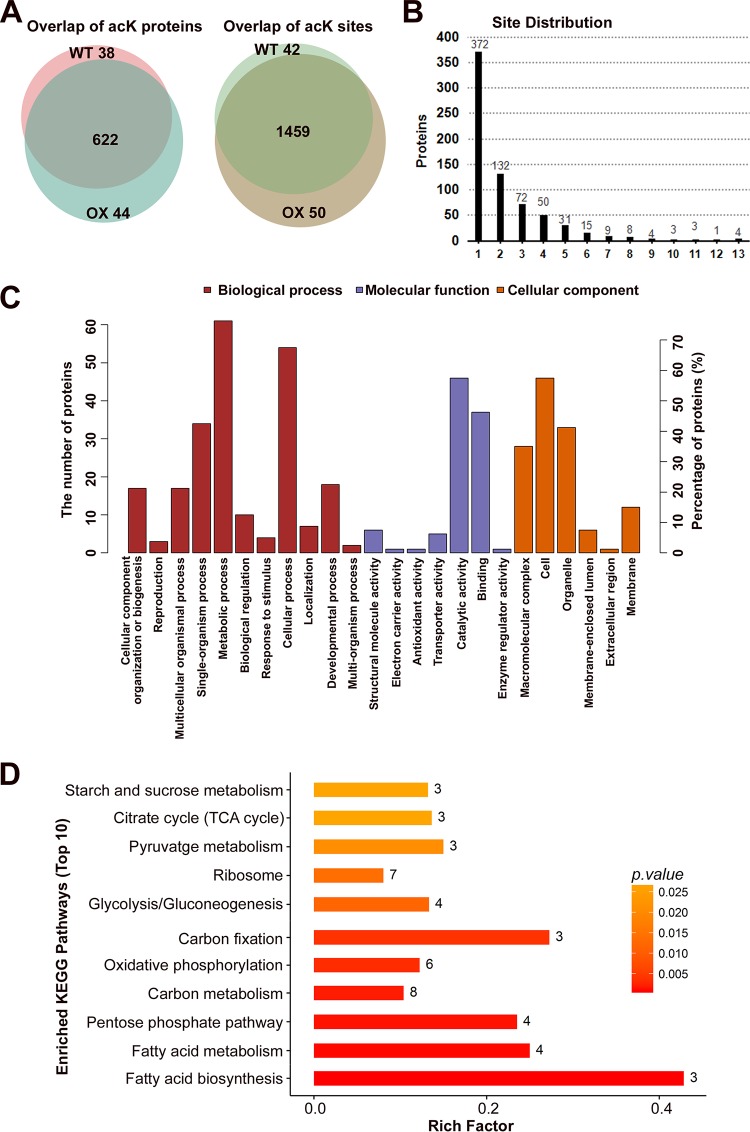
Identification of acetylated peptides and proteins by LC-MS/MS. (A) Overlap of identified acetylated proteins and peptides from WT and OX mycelia. (B) Distribution of acK sites identified per protein. The numbers of proteins with different numbers of acetylation sites (1 to 13) are shown above the bars. (C) Gene Ontology (GO) term (level 2) distribution for the acetylome of WT and OX mycelia. The proteins were classified by GO annotation based on three categories: biological process, cellular component, and molecular function. (D) KEGG pathway-based enrichment analysis of the acetylated proteins. The KEGG pathway database (http://www.genome.jp/kegg/pathway.html) was used to annotate the pathways of the differentially acetylated proteins.

By using a false discovery rate (FDR) cutoff of less than or equal to 1%, we identified 1,551 unique AcK sites across 704 proteins (see Data Set S1 in the supplemental material), with an overlap of 94% and 93%, respectively, between the WT and OX (Fig. 1A). Almost half of these 704 proteins were multiacetylated, with 47% having two or more AcK sites (Fig. 1B). The most acetylated proteins were the Hsp70-like protein (MGG_06958), heat shock protein 90 (Hsp90; MGG_06759), glyceraldehyde-3-phosphate dehydrogenase (G3PD; MGG_01084), and an uncharacterized protein (MGG_11259), with 13 acetylated lysines, respectively. We also found abundant acetylation on histone proteins (Data Set S1), including H2A.Z (5 of 18 lysines acetylated), H3 (8 of 14 lysines acetylated), H2A (5 of 12 lysines acetylated), H2B (12 of 23 lysines acetylated), H4 (MGG_06093; 1 of 12 lysines acetylated), H3-like centromeric protein cse-4 (1 of 12 lysines acetylated), and H4 (MGG_01160; 6 of 11 lysines acetylated). This confirmed the role of Gcn5 as a chromatin modifier that epigenetically regulates global gene transcription through modification on histone proteins in *M. oryzae*. However, most of these proteins or lysine residues did not show significant differences in acetylation levels between the OX and WT strains, and therefore are unlikely to be the specific substrates of Gcn5.

10.1128/mSystems.00270-18.5 DATA SET S1 Acetyl proteins/residuals identified by acetylome analysis. Download Data Set S1, XLS file, 1.9 MB. Copyright © 2018 Liang et al. 2018 Liang et al. This content is distributed under the terms of the Creative Commons Attribution 4.0 International license.

### Identification and enrichment of lysine acetylation sites potentially dependent on Gcn5.

To identify true substrates of Gcn5 among all acetylated proteins and sites, we compared the relative levels of AcK peptide abundances between WT and OX mycelia using label-free quantitation. Of the 1,551 acK sites identified, we found that acetylation at 92 sites present in 82 proteins changed more than twofold (*P* ≤ 0.05) in OX mycelia at an FDR of 0.04 (Data Set S2).

10.1128/mSystems.00270-18.6 DATA SET S2 Differential acetyl proteins/residuals identified by label-free quantification. Download Data Set S2, XLSX file, 0.4 MB. Copyright © 2018 Liang et al. 2018 Liang et al. This content is distributed under the terms of the Creative Commons Attribution 4.0 International license.

Gene Ontology (GO) annotation and enrichment with the acetylated proteins significantly different between the WT and OX samples were as listed in Data Set S3 and schematically represented in three ontologies as molecular function, cellular component, and biological process, as in Fig. 1C. KEGG pathway annotation and enrichment were as listed in Data Set S4 and schematically represented as in Fig. 1D. These pathway analyses revealed the enrichment of differentially acetylated proteins involved in carbon metabolism, including glycolysis, pentose phosphate pathway, starch and sucrose metabolism, TCA cycle, and carbon fixation pathway, in the OX samples compared to the WT samples. The second enriched pathway in the OX samples was the fatty acid metabolism and fatty acid degradation pathway. Another group of enriched proteins in the OX samples were shown to be involved in the signaling pathway of mitochondrial oxidative phosphorylation, which is likely leading to programmed cell death (24). Thus, *GCN5OX* mycelia show a profound altered protein acetylation across metabolic and signaling pathways.

10.1128/mSystems.00270-18.7 DATA SET S3 GO annotation and enrichment with the acetyl proteins significantly different between the WT and the OX samples. Download Data Set S3, XLS file, 2.0 MB. Copyright © 2018 Liang et al. 2018 Liang et al. This content is distributed under the terms of the Creative Commons Attribution 4.0 International license.

10.1128/mSystems.00270-18.8 DATA SET S4 KEGG pathway annotation and enrichment with the acetyl proteins significantly different between the WT and OX samples. Download Data Set S4, XLSX file, 0.04 MB. Copyright © 2018 Liang et al. 2018 Liang et al. This content is distributed under the terms of the Creative Commons Attribution 4.0 International license.

### Gcn5 regulates *M. oryzae* autophagy induction via histone and nonhistone protein acetylation.

Because there have been limited reports of substrates and sites of Gcn5 regulation, we compared our data with the data previously reported for histone H3. There were 8 acetylated lysine residues detected in H3, namely, H3 K9, K14, K18, K23, K27, and K36 as reported (25, 26), and H3 K4, K56, and K79 that have not been reported in other organisms. No change was detected between the WT and OX strains in the AcK levels of most of the H3 acetylated lysines, except for H3 K79, which was absent in the OX strain (Fig. S2A and Data Set S2). We inferred that phototropic induction of H3 acetylation may reach a saturated level; therefore, overexpression of *GCN5* could not further increase H3 acetylation, at most of the AcKs. H3K79 acetylation may be catalyzed by another histone acetyltransferase, whose function may be suppressed by Gcn5; thus, H3K79ac was lost in the OX strain. To further confirm this hypothesis, a single set of acetylome analysis using OX and KO (*gcn5*Δ) mutants (Data Set S5) also identified multiple acetylation sites, and H3 K79 showed higher acetylation in the KO mutant compared to the OX mutant.

10.1128/mSystems.00270-18.2 FIG S2 Acetylation profiles of histone H3 and Atg7 and PK expression assessed by qRT-PCR. (A) Acetylation profiles of histone H3. (B) Acetylation profiles of Atg7. **, significant difference (*P* < 0.05). (C) qRT-PCR to assess expression of WT or mutated forms of PK. Relative fold changes were calculated using the −ΔΔCt method. The *ACTIN* gene (*MGG_03982*) was used as an internal control. Asterisks denote significant difference (*P* < 0.05) compared to the WT values. There were no significant differences between the values for the three overexpression strains. Download FIG S2, TIF file, 0.4 MB. Copyright © 2018 Liang et al. 2018 Liang et al. This content is distributed under the terms of the Creative Commons Attribution 4.0 International license.

10.1128/mSystems.00270-18.9 DATA SET S5 Differential acetyl proteins identified in KO versus OX samples and Gcn5-associated proteins identified by LC-MS/MS. Download Data Set S5, XLSX file, 0.5 MB. Copyright © 2018 Liang et al. 2018 Liang et al. This content is distributed under the terms of the Creative Commons Attribution 4.0 International license.

**FIG 2:**
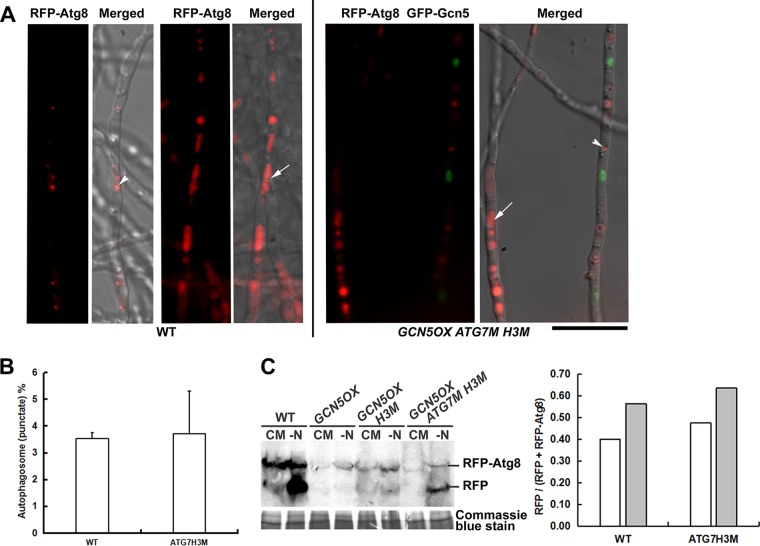
Epigenetic and posttranslational regulation of nitrogen-induced autophagy in *M. oryzae*. (A) Autophagic activity visualized by RFP-Atg8 signal under nitrogen starvation. Representative images for WT or *GCN5OX ATG7M H3M* mycelia are presented. Vacuolar RFP signal (white arrows), autophagosome (white arrowheads) are indicated. Bar = 5 μm. (B) Quantification of autophagy activity by calculating the percentage (mean ± standard error [SE]) of mycelial area labeled by RFP-Atg8 as autophagosomes or autophagic vacuoles by using software ImageJ (https://imagej.nih.gov/ij/) and the “Analyze>Analyze Particles” function. More than 10 pieces of mycelia were used for such quantification in each instance. (C) Total protein lysates from WT, *GCN5OX*, *GCN5OX H3M*, and *GCN5OX ATG7M H3M* strain were analyzed by immunoblotting with anti-RFP antibodies (rabbit; 1:1,000; Clontech, catalog no. R10367). The presence of free RFP band indicates induction of autophagy. The extent of autophagy was estimated by calculating the amount of free RFP compared with the total amount of intact RFP-Atg8 and free RFP and presented in the bar chart for WT and *GCN5OX ATG7M H3M* strains. Densitometric analysis was performed by ImageJ (https://imagej.nih.gov/ij/). Coomassie blue stain of the total protein lysates serves as a loading control.

**FIG 3:**
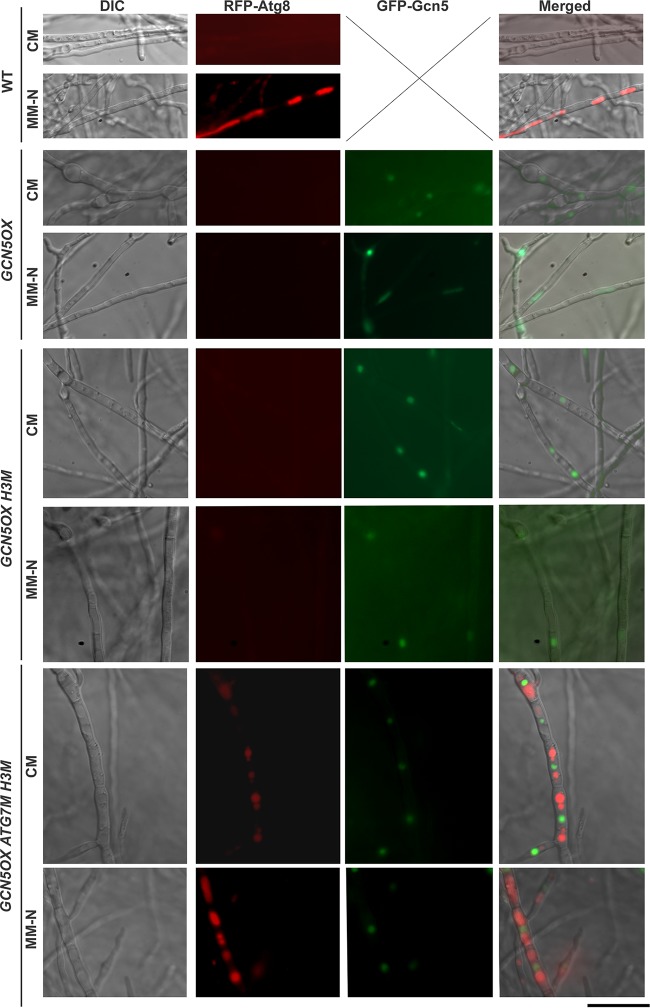
Epigenetic and posttranslational regulation of nitrogen-induced autophagy in *M. oryzae*. Autophagic activity visualized by RFP-Atg8 signal in CM- or MM-N-cultured mycelia of the WT, OX, OX (H3M). and OX (Atg7M H3M) mutants. GFP-Gcn5 signal appeared in the strains carrying the overexpressed *GCN5* gene. DIC, differential interference contrast. Bar = 5 μm.

Consistent with our previous finding (21), an autophagy protein Atg7 was found to be acetylated at two sites, K107 and K338. Atg7 K338ac was significantly higher in the OX than in the WT, while K107ac was changed slightly (Fig. S2B and Data Set S2). This result confirmed that Atg7 K338 acetylation is Gcn5 dependent. However, our point mutation of Atg7 (K338R) could not restore phototropic induction of autophagy in the OX strain (unpublished data). It has been reported that histone H3 hyperacetylation in Drosophila melanogaster is associated with transcriptional downregulation of several autophagy-essential *ATG* genes (*ATG5*, *ATG7*, and *ATG14*), which may restrict autophagic activities (27–29). A recent report displayed that deacetylation of H3 may increase autophagy genes *ATG6*, *ATG15*, and *ATG16* and *ATG22* expression and thus also modulate *M. oryzae* autophagy (16). As mentioned above, H3 acetylation in OX was at a comparable level with WT, for most of the detected H3 acK sites, except for H3 K79. Therefore, we designed and expressed H3 K79Q (acetylation mimic) mutation in the *GCN5OX* strain, alone or together with the Atg7 K338R mutation. Interestingly, we observed that a combination of H3 K79Q and Atg7 K338R mutation could successfully restore autophagy induction in the *GCN5OX* mutant in response to nitrogen starvation, while either single point mutations could not (Fig. 2A to C and Fig. 3). We therefore conclude that Gcn5 regulates *M. oryzae* autophagy induction at both the epigenetic (through H3 modification) and posttranslational (Atg7 modification) levels.

### Gcn5 regulates pyruvate kinase protein stability via acetylation.

Our comparative acetylome analysis also identified that lysine 188 (K188) on pyruvate kinase (PK), the key enzyme of glycolysis, was slightly more acetylated in the OX strain than in the WT strain (Data Set S2), while it was significantly reduced in the KO strain (Data Set S5), and was among Gcn5-associated proteins isolated by immunoprecipitation and identified also by LC-MS/MS (Data Set S5). The GFP-Gcn5 strain we used in immunoprecipitation is the same OX strain used in our comparative acetylome analysis.

**FIG 4:**
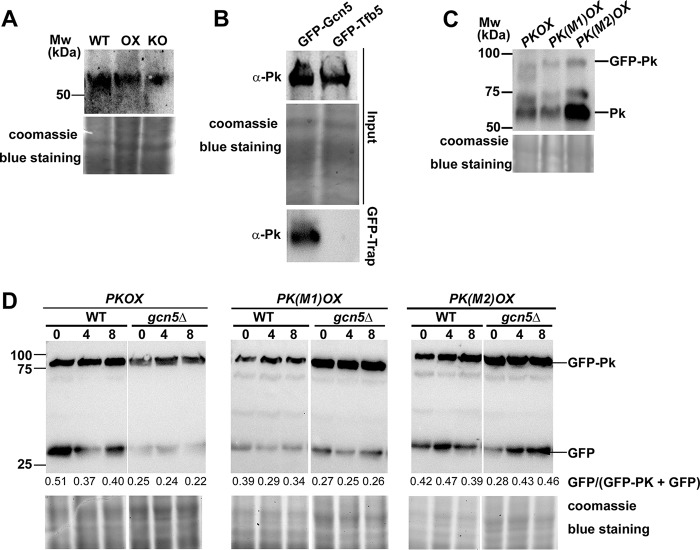
Gcn5 interacts with Pk and may be responsible for Pk stability. (A) Total protein lysates were extracted from wild-type (WT), *GCN5OX* (OX), and *gcn5*Δ (KO) strains, and then analyzed by immunoblotting with anti-PK antibodies (rabbit; 1:500; Abcam, ab34554). Detection of a single band higher than the 50-kDa molecular marker band corresponds to Pk protein. Coomassie blue staining of SDS-PAGE of total proteins served as a loading control. (B) Coimmunoprecipitation assays among Gcn5 and Pk proteins. The *M. oryzae* strains carrying GFP-Gcn5 or GFP-Tfb5 were grown in liquid CM at 28°C for 48 h. Total proteins extracted from the strains were subjected to immunoprecipitation with GFP-Trap (Chromo Tek, gta-20), and the IP proteins were separated by 12% SDS-PAGE and detected by anti-Pk antibody (α-Pk). Coomassie blue staining of SDS-PAGE of total proteins served as a loading control for input proteins. (C) Total protein lysates were extracted from the *M. oryzae* strains carrying *PKOX*, *PK*(*M1*)*OX*, and *PK*(*M2*)*OX* and then analyzed by immunoblotting with anti-PK antibodies. The fused GFP-Pk protein and the Pk peptide alone were detected, judged by the molecular weight (Mw) as shown. Coomassie blue staining of SDS-PAGE of total proteins served as a loading control. (D) Total protein lysates were extracted from the *M. oryzae* strains carrying *PKOX*, *PK*(*M1*)*OX*, and *PK*(*M2*)*OX*, in both WT or *gcn5*Δ background, in a time course of 0, 4, and 8 h after treatment with the protein synthesis inhibitor cycloheximide (Sigma-Aldrich, catalog no. C104450). Detection of the fused GFP-Pk protein and GFP peptide alone was labeled. The extent of Pk degradation was estimated by calculating the amount of free GFP compared with the total amount of intact GFP-Pk and free GFP (the numbers appear below the blot). Densitometric analysis was performed using ImageJ (https://imagej.nih.gov/ij/). Coomassie blue staining of SDS-PAGE of total proteins served as a loading control.

**FIG 5:**
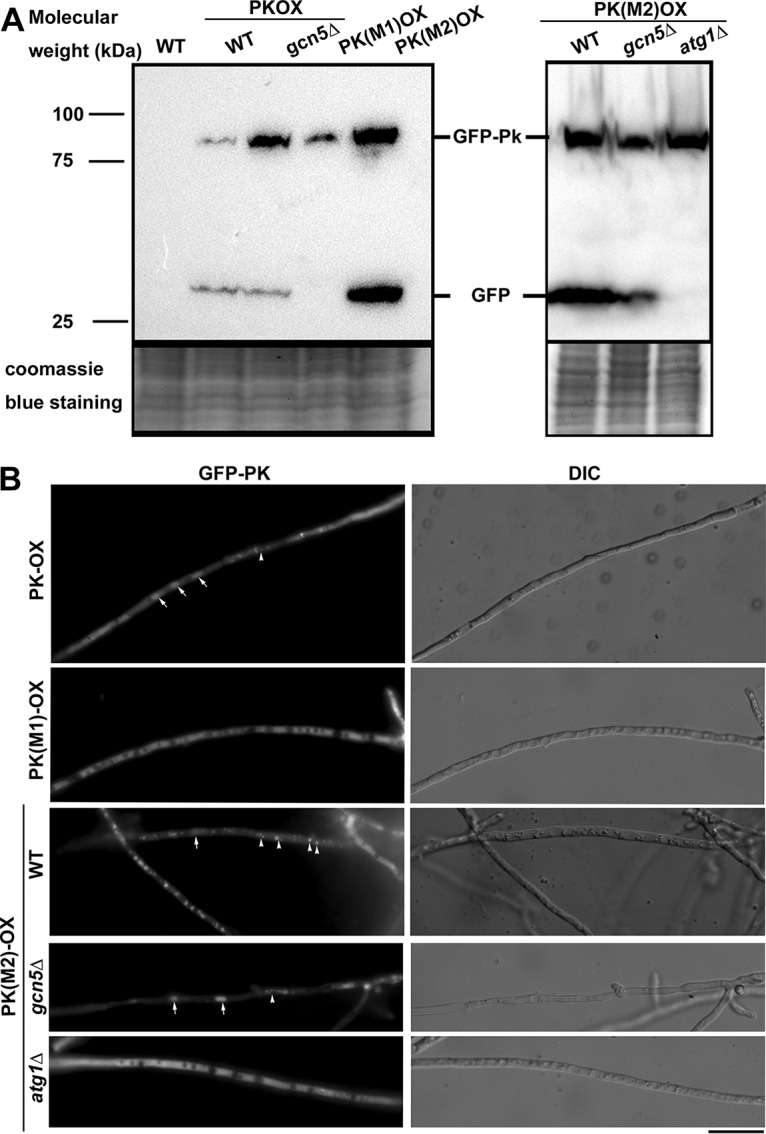
Gcn5-mediated acetylation on Pk lysine 188 may contribute to autophagy degradation of Pk. (A) Total protein lysates were extracted from the wild-type (WT) strain, three versions of *PK*-overexpressed strains in the WT background, *PKOX* in the *gcn5*Δ background, and WT, *gcn5*Δ, and *atg1*Δ (autophagy-deficient) strains expressing M2 (K188Q) version of *PK*. Total protein lysates were analyzed by immunoblotting with anti-GFP antibodies (rabbit; 1:5,000; Invitrogen Molecular Probes, catalog no. A6455). The presence of the free GFP band indicates cleavage between GFP and Pk protein, and likely degradation of fusion proteins. Coomassie blue staining of SDS-PAGE of total proteins served as a loading control. (B) Epifluorescence microscopy with *PKOX*, *PK*(*M1*)*OX* (K188R, unacetylated mimic) strain, and WT, *gcn5*Δ, and *atg1*Δ (autophagy-deficient) strains expressing M2 (K188Q) version of *PK*. Arrows denote spherical vacuoles and arrowheads indicate vesicular puncta or filamentous vacuoles. Bar = 5 μm.

First, we verified that the elevated acetylation level of Pk protein in the OX strain compared to the level in the WT strain did not result from the potential increased Pk protein. Using anti-Pk antibody (Abcam, ab34554), we detected the endogenous Pk level in the WT, OX, and KO strains, and our results showed no obvious difference in the Pk protein level in these three strains (Fig. 4A), confirming that Pk acetylation was indeed increased with *GCN5* overexpression. Next, we verified the direct interaction between Gcn5 and Pk by pulling down the GFP-Gcn5-associated proteins using GFP-Trap kit (Chromo Tek, gta-20) and detecting the presence of Pk protein using anti-Pk antibody. Our results showed that Pk was detected in the GFP-trapped samples from the GFP-Gcn5 (OX) strain while absent in that from Tfb5-GFP strain (serving as a negative control; Fig. 4B). Overall, we inferred that Pk may be a potential substrate of Gcn5 and may be possibly related to *M. oryzae* carbon metabolism. As the *PK* gene encodes a critical enzyme in glycolysis and may be essential for cell viability, we were unable to obtain deletion mutants of it, although repeated attempts have been made. To investigate its function, we overexpressed this gene in the WT form and two mutation forms, K188R (M1, unacetylable) and K188Q (M2, acetylated mimic), respectively, in the wild-type *M. oryzae*. The transcription level of *PK* in the wild-type *M. oryzae* and in three forms of *PK* overexpression strains was verified by qRT-PCR (Fig. S2C). The fused GFP-Pk protein (WT or mutated varieties) was detected by anti-PK antibody (Fig. 4C). To our surprise, at the protein level, the WT and M1 forms of Pk seemed more stable than the M2 form of Pk, as an increase in the Pk peptide alone was detected in the M2 mycelia, indicative of enhanced degradation of GFP-Pk fusion protein (Fig. 4C). Similarly, when we detected the fusion protein with anti-GFP antibody, we also observed a higher level of GFP peptide accumulation in the M2 mycelia (Fig. 5A). When expressed in the *gcn5*Δ background, the wild-type GFP-Pk fusion protein seemed more abundant than in the WT background, indicating that loss or reduced Pk acetylation mediated by Gcn5 may stabilize Pk. In other word, acetylated Pk (likely on K188) is subject to degradation.

To verify that Gcn5-mediated Pk acetylation contributes to Pk degradation, we performed a time course detection of cleavage of GFP peptide from the GFP-Pk fusion protein in these three overexpressed Pk varieties in both the WT or *gcn5*Δ background. Our results showed that cleavage of GFP peptide from the GFP-Pk fusion protein, indicative of GFP-Pk degradation, was obviously suppressed in the *PK*(*M1*)*OX* (K188R) mutant, but not in the *PK*(*M2*)*OX* (K188Q) mutant, and such degradation could be effectively suppressed with the loss of *GCN5* in the *PKOX* strain (Fig. 4D). We observed that cleavage of GFP-Pk(M2) (K188Q, acetylation mimic) was comparable in the WT or *gcn5*Δ background, further confirming a role of acetylation in Pk degradation.

Interestingly, we found that such degradation of the GFP-Pk(M2) protein (K188Q mutation) was completely lost in the autophagy-deficient mutant *atg1*Δ background, but not in the *gcn5*Δ background (Fig. 5A). Correspondingly, we observed that the M2 mutated form of GFP-Pk fusion protein accumulated in vacuoles of *M. oryzae* mycelia, while the M1 (unacetylated mimic) form of GFP-Pk was cytosolic (Fig. 5B). Vacuolar localization of M2 GFP-Pk was lost in the *atg1*Δ mutant, but not in the *gcn5*Δ mutant (Fig. 5B).

Overall, we infer that Gcn5 may catalyze acetylation on Pk lysine 188, which facilitates its degradation in an autophagy-dependent manner. This observation is consistent with a recent report for cancer cells that acetylation of the embryonic M2 isoform of pyruvate kinase (named PKM2) promotes its degradation via chaperone-mediated autophagy, which links metabolic shift and tumor growth (30). However, unlike in animal cells, *M. oryzae* has only one Pk protein but not with any other isoform. By acetylation- and autophagy-dependent degradation of the only Pk protein, *M. oryzae* could modulate its carbon metabolism (glycolysis) and likely also determine its cell fate.

### Pyruvate kinase plays an important role in *M. oryzae* pathogenicity.

**FIG 6:**
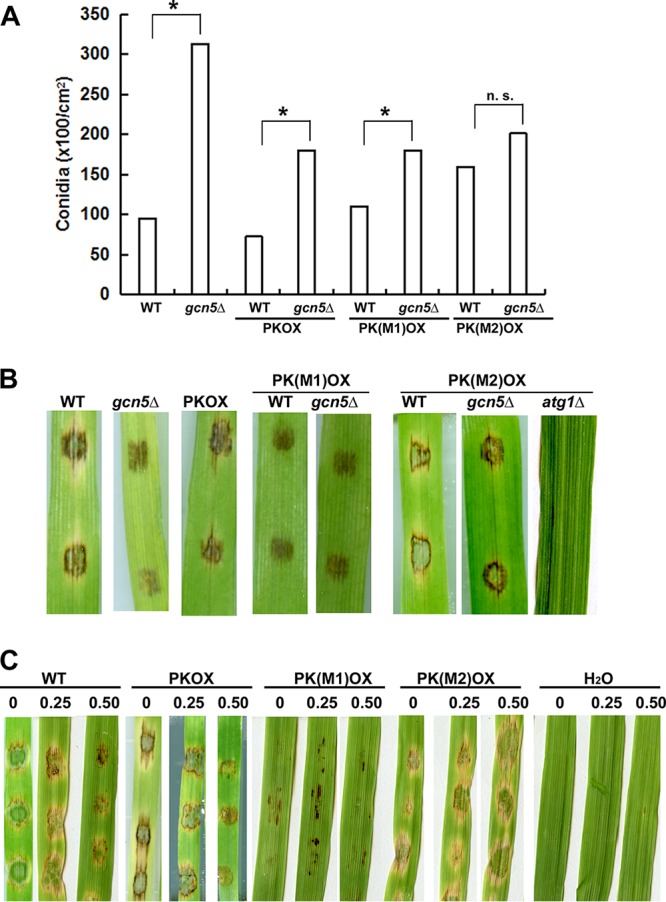
Characterization of mutant strains carrying overexpressed *PK* variants. (A) Bar chart depicting quantitatively assessed conidiation in the *PKOX* variants compared with the WT strain, grown on prune agar (PA) medium. Mean values (±SE) were derived from three independent experiments (*n* = 4 colonies for each sample). Assessments were performed 3 days after photoinduction. n. s., not significant. (B) Disease symptoms on cut leaves of barley inoculated with WT (B157), *gcn5*Δ mutant, *PKOX*, and *PK*(*M1*)*OX*, *PK*(*M2*)*OX* conidia in WT and different mutant backgrounds, respectively, were photographed at 7 day postinoculation. (C) Disease symptoms on cut leaves of barley inoculated with WT (B157), *PKOX*, *PK*(*M1*)*OX*, and *PK*(*M2*)*OX* conidia with or without pyruvate at different concentrations (labeled on top of the images of inoculated leaves), were photographed at 7 days postinoculation. Inoculation with water (H_2_O) with 0.25 and 0.50 mM pyruvate on barley leave explants served as a negative control.

We further investigate the biological significance of Pk acetylation (and degradation) in *M. oryzae* by characterizing conidiation (production of asexual spores) and infection of the strains carrying these overexpressed *PK* variants. For conidiation, we observed no obvious difference between the wild-type (WT) strain and the *PKOX* variants (*P* > 0.05; Fig. 6A), except for the *PK*(*M2*)*OX* mutant (K188Q mutation; *P* < 0.05). This suggests that Pk protein acetylation may promote *M. oryzae* conidiation. We have reported that the *gcn5*Δ mutant produced more conidia than the wild-type strain did, likely due to promotion of autophagy induction (21), and here we further assessed conidiation of the *gcn5*Δ mutant for each *PKOX* variant. We found that as in the wild-type background, deletion of the *GCN5* gene in the *PKOX* strain or in the *PK*(*M1*)*OX* strain (K188R mutation) resulted in a hyperconidiation phenotype (*P* < 0.05; Fig. 6A), while such hyperconidiation was not observed in the *PK*(*M2*)*OX* mutant (K188Q mutation; *P* > 0.05; Fig. 6A). This result could be viewed as an indication that the overexpressed wild-type form or the R or Q mutation of Pk variants is functional. We further infer that both autophagy induction and Pk acetylation play a positive role in *M. oryzae* conidiation and are subject to Gcn5 regulation.

More interestingly, infection on the barley leaf explants with *PK*(*M1*)*OX* conidia was significantly reduced compared to that of WT conidia, and similar to that of the *gcn5*Δ conidia (Fig. 6B). Our previous study reported that Gcn5 is required for full pathogenicity, but the mechanism is not fully understood (21). Here we added a piece of evidence that the pathogenicity defect of the *gcn5*Δ mutant may due to loss/reduced acetylation on Pk protein, which leads to stabilization of this protein. To further support our hypothesis, the *PK*(*M2*)*OX* conidia in the *gcn5*Δ background could partially restore its pathogenicity, but not in the *atg1*Δ (autophagy-deficient) background (Fig. 6B). This result demonstrates that autophagy-dependent degradation of acetylated Pk is crucial for *M. oryzae* pathogenicity. Therefore, our study identified a Gcn5 substrate, Pk, with biological significance in *M. oryzae* pathogenicity.

As Pk acetylation was reported to be responsible for a metabolic shift in cancer cells (30), we suspected that the pathogenicity defect in the *PK*(*M1*)*OX* or *gcn5*Δ mutant may result from excessive production of pyruvate, the direct product of Pk-catalyzed glycolysis, as a consequence of the loss of Pk acetylation (on lysine 188 residue) and thus, block/delay Pk degradation via the autophagy pathway. To test this hypothesis, we assessed the effect of pyruvate on *M. oryzae* pathogenicity by inoculating conidia from the WT strain and the strain carrying overexpressed *PK* variants, with or without pyruvate, on barley leaf explants. We observed that with increased concentrations of pyruvate, the pathogenicity of WT or *PKOX* conidia gradually decreased (Fig. 6C). *PK*(*M1*)*OX* conidia were very weak in pathogenicity with or without pyruvate treatment, while *PK*(*M2*)*OX* conidia remained pathogenic even in the presence of 0.50 mM pyruvate (Fig. 6C). Furthermore, when inoculated on an artificial surface, 0.5 mM pyruvate could effectively suppress appressorium formation in the wild-type strain and in the three *PK* overexpression mutants, while 0.25 mM pyruvate could not (Table 1). Pyruvate was shown to be essential for *M. oryzae* appressorium formation and pathogenicity, and it could be produced as an intermediate of the glyoxylate cycle by a peroxisomal alanine:glyoxylate aminotransferase, Agt1 (31). Here our infection assay revealed that excessive pyruvate could also suppress *M. oryzae* appressorium formation and pathogenicity, and therefore, the endogenous pyruvate level needs to be fine-tuned. Besides Agt1, Pk is another critical enzyme controlling pyruvate production and therefore, it plays an important role in *M. oryzae* pathogenicity.

**TABLE 1:** Pyruvate suppressed *M. oryzae* appressorium formation

Strain	AP%^*a*^		
	Untreated	0.25 mM pyruvate	0.50 mM pyruvate
Wild type	87.2 ± 3.5	90.65 ± 9.2	7.5 ± 0.7**
PKOX	91.5 ± 9.2	74.3 ± 17.1	13.1 ± 8.5**
PK(M1)OX	94.7 ± 3.4	91.6 ± 3.5	18.0 ± 24.1**
PK(M2)OX	92.0 ± 5.6	87.5 ± 13.4	53.0 ± 16.8**

a AP%, percent appressorium formation. The values are means ± standard errors (SE) derived from three independent repeats (*n* ≥ 50 for each instance). Values for pyruvate-treated conidia that are significantly different from the values for the untreated condition for the same strain are indicated by a pair of asterisks.

### Gcn5 regulates gene transcription.

As Gcn5 also modulates (directly or indirectly) histone protein H3’s acetylation, we infer that Gcn5 could also epigenetically regulate global gene transcription in *M. oryzae*. Therefore, we performed transcriptome analysis with OX samples versus WT samples under the same conditions as we performed comparative acetylome analysis to identify potential target genes subject to epigenetic regulation by Gcn5 function. In total, we identified 147 differentially expressed genes (DEGs) (absolute log_2_ fold change of >1 and FDR of ≤0.05), 60 upregulated genes and 87 downregulated genes (Data Set S6). Among the DEGs listed in Data Set S6, we noticed that genes encoding components of stress response, particularly oxidative stress response (*MGG_03329*, *MGG_05719*, *MGG_09226*, *MGG_10760*, *MGG_10239*, *MGG_01941*, *MGG_09162*, and *MGG_09212*), carbohydrate/fatty acid/amino acid metabolism (*MGG_09190*, *MGG_17103*, *MGG_03995*, and *MGG_08537*), membrane transporters (*MGG_09680*, *MGG_15435*, *MGG_15331*, *MGG_15411*, *MGG_09119*, *MGG_01864*, *MGG_04248*, *MGG_04864*, *MGG_14824*, and *MGG_05880*), transcriptional factors (*MGG_10575*, *MGG_08536*, and *MGG_09273*), cell wall biosynthesis (*MGG_17153*, *MGG_08527*, and *MGG_07677*), mycelial/conidial pigmentation (*MGG_07219* and *MGG_05059*), and secondary metabolism (*MGG_00806*, *MGG_10671*, and *MGG_08527*), were upregulated in the OX samples. *GCN5* overexpression was also verified by the transcriptome analysis, as it (*MGG_03677*) was found among the upregulated genes (Data Set S6; 2.6-fold, *P* ≈ 0.0005). In addition, we were interested in the finding that a bacteriorhodopsin-like protein-encoding gene (*MGG_09015*) was upregulated in the OX strain, as bacteriorhodopsin serves as light-driven proton pump in halobacteria, eubacteria, or fungi (32–34). Given that the functioning photosensor or photoreceptor for phototropic conidiation has not yet identified, this bacteriorhodopsin-like protein may be a potential candidate. In the list of downregulated genes, we also found abundant genes encoding transcriptional factors/regulators (*MGG_13350*, *MGG_04213*, *MGG_13360*, *MGG_04171*, *MGG_06312*, and *MGG_10422*), membrane transport proteins (*MGG_03341*, *MGG_10893*, *MGG_04234*, *MGG_07639*, and *MGG_1086*9), enzymes involved in cellular metabolism (*MGG_10023*, *MGG_09150*, *MGG_12228*, *MGG_03392*, *MGG_01924*, *MGG_10274*, *MGG_04345*, *MGG_01925*, *MGG_07626*, *MGG_13239*, *MGG_09994*, *MGG_09647*, *MGG_08072*, *MGG_00311*, *MGG_02261*, and *MGG_08989*), and enzymes involved in cell wall biosynthesis (*MGG_14966*, *MGG_09167*, *MGG_07214*, *MGG_06585*, and *MGG_00110*). A serine/threonine protein kinase encoded by *MGG_11636* was found to be downregulated in the OX, which is the only kinase identified to be differentially regulated by *GCN5* in our transcriptome analysis. A SET protein-encoding gene (*MGG_00019*) was repressed by *GCN5*, which is reasonable, as SET proteins inhibit acetylation of nucleosomes by histone acetylases (HAT), especially on histone H3 and H4 (35, 36). Vegetative (heterokaryon) incompatibility responses, which usually result in cell death, may also be suppressed by *GCN5*, probably via downregulation of *MGG_09107*, encoding a heterokaryon incompatibility protein. Correlating with this aspect, genes whose products may be involved in cell toxicity and/or death, including a prion inhibition and propagation protein-encoding gene (*MGG_00203*), an NACHT domain-containing protein (*MGG_09355*), a BRCT domain-containing protein (*MGG_01032*), were all repressed by *GCN5*. However, three autophagy genes, *ATG5* (*MGG_09262*), *ATG7* (*MGG_07297*), and *ATG14* (*MGG_03698*), were not among the DEGs between WT and OX strains under photoinduction. Overall, our transcriptome analysis reveals *GCN5-*dependent regulation, either directly or indirectly, of global transcription of genes involved in cellular metabolism, stress response, cell toxicity, and death.

10.1128/mSystems.00270-18.10 DATA SET S6 Differentially expressed genes (DEGs) between the WT and OX samples identified by transcriptome analysis. Download Data Set S6, XLS file, 0.08 MB. Copyright © 2018 Liang et al. 2018 Liang et al. This content is distributed under the terms of the Creative Commons Attribution 4.0 International license.

**FIG 7:**
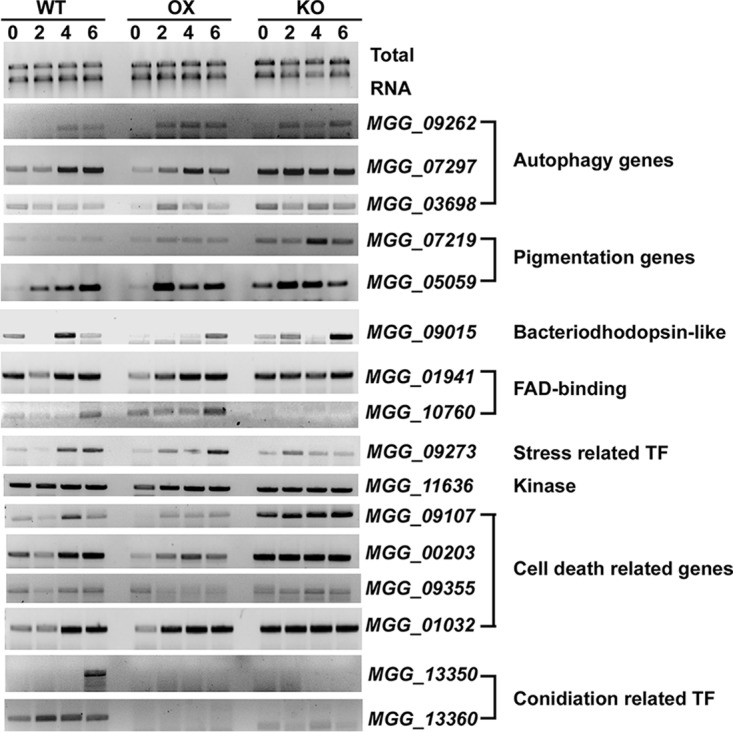
Transcriptional profiling of selected genes by RT-PCR. Total RNA was extracted from the wild-type (WT), *GCN5OX* (OX), or *gcn5*Δ (KO) mycelia exposed to light for 0, 2, 4, and 6 h and used for RT-PCR analysis with selected candidate genes. A 1% denaturing agarose gel of total RNA showing 18S and 28S rRNA served as a loading control. The experiments were repeated three times, and representative results are displayed here. TF, transcription factor.

A subset of candidate genes from the DEGs were selected for further verification. The list of selected genes and the respective primer sequences are shown in Table S1. We noticed that two FAD-binding protein-encoding genes (*MGG_07219* and *MGG_05059*) were upregulated in the OX (*GCN5OX*) strain compared to the WT (Fig. 7), indicating that the OX strain may be more resistant/tolerant to the oxidative stress, which is consistent with the sensitivity test results reported previously (21). Also, a stress-responsive transcriptional factor, *MGG_09273*, was upregulated in the OX strain while reduced in the KO (*gcn5*Δ) mutant (Fig. 7). Pigmentation occurs during *M. oryzae* conidiation and infection (37), and two pigment-related genes (*MGG_07219* and *MGG_05059*) were identified as upregulated in the OX strain (Data Set S6) and were verified by RT-PCR (Fig. 7). A gene encoding a bacteriorhodopsin-like protein (*MGG_09015*) was found highly upregulated in the OX strain compared to the WT strain (Data Set S6). Surprisingly, we detected even higher expression levels of this gene in the KO strain than in either the WT or OX strain (Fig. 7). It is worth following up to confirm the expression pattern of this gene, and more importantly, the functional analysis of this gene as a potential light sensor involved in phototropism of *M. oryzae*. For the observation that some candidate genes, including two pigmentation genes and a gene encoding bacteriorhodopsin-like protein were upregulated in the OX strain compared to the WT strain, while they were upregulated to an even greater extent in the KO strain, we do not know the exact reason at present. The HAT Gcn5 may regulate gene expression in both a direct manner (via acetylating histone proteins) or indirect manner (via acetylating other HAT or HDAC enzymes and modulating their activity and thus yielding an effect on gene expression through other histone modifiers). Therefore, overexpression of Gcn5 may result in upregulation of gene expression, while deletion of Gcn5 may change the activity of another histone modifier(s) as a nonhistone substrate(s) of Gcn5, and may also lead to induction of the same set of genes.

10.1128/mSystems.00270-18.3 TABLE S1 Selected differentially expressed genes for verification by RT-PCR. Download Table S1, DOCX file, 0.02 MB. Copyright © 2018 Liang et al. 2018 Liang et al. This content is distributed under the terms of the Creative Commons Attribution 4.0 International license.

It was also interesting to observe that four genes (*MGG_09107*, *MGG_00203*, *MGG_09355*, and *MGG_01032*) involved in cell death were all significantly downregulated in the OX strain (Data Set S6). Our RT-PCR verified that *MGG_09107*, *MGG_00203*, and *MGG_09355* were indeed expressed at lower levels in the OX strain, while they were expressed at higher levels in the KO strain than in the WT strain (Fig. 7). However, the differential expression level of *MGG_01032* was not prominent in these three strains (Fig. 7). Transcription of autophagy genes *ATG5*, *ATG7*, and *ATG14* were comparable between the OX and WT strains as verified by RT-PCR (Fig. 7). We could not rule out the possibility that epigenetic regulation of transcription of another autophagy gene(s) contributes to regulation of autophagy in *M. oryzae*. Overall, we verified that Gcn5 is indeed involved in epigenetic regulation of several candidate genes involved in cellular development and/or cell and death, as well as stress response, likely via histone protein acetylation. However, the exact function of such candidate genes and the regulatory mode by Gcn5 await further investigation.

### Sequence motif analysis of Gcn5-regulated acetylation sites in *M. oryzae*.

To determine whether there is a common sequence motif required for acetylation by Gcn5, we compared the amino acid sequences of all significantly increased acetylated sites in the OX strain by using Motif-x (38) (Table S2). A preference was observed for leucine, phenylalanine, aspartic acid, or glutamic acid at the −1 position and for leucine, phenylalanine, arginine or lysine at the +1 position. Interestingly, Gcn5 substrates showed a stronger preference for K at the +1 and +4 positions (Table S2).

10.1128/mSystems.00270-18.4 TABLE S2 Statistical analysis of the acK motif significantly increased in the OX strain. Download Table S2, DOCX file, 0.2 MB. Copyright © 2018 Liang et al. 2018 Liang et al. This content is distributed under the terms of the Creative Commons Attribution 4.0 International license.

**FIG 8:**
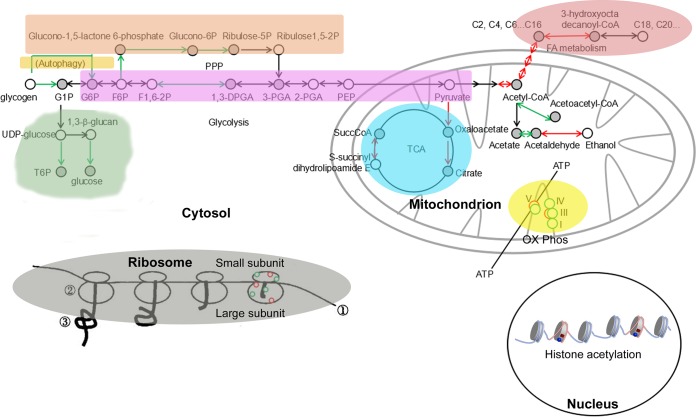
Schematic depicting the Gcn5-regulated metabolic pathways. Circles represent constituent proteins or protein complexes within each pathway. Abbreviations: G1P, glucose 1-phosphate; G6P, glucose 6-phosphate; F1,6-2P, fructose 1,6-bisphosphate; 1,3-DPGA, 1,3-bisphosphoglyceric acid; 2-PGA, 2-phosphoglyceric acid; PEP, phosphoenolpyruvic acid; T6P, trehalose phosphate; SuccCoA, succinyl-CoA; OX Phos, oxidative phosphorylation; FA, fatty acid; TCA, tricarboxylic acid.

The histone acetyltransferase (HAT) Gcn5 was shown to be essential for regulating *M. oryzae* conidiation and pathogenicity (21), but the underlying mechanism, which may be pleiotropic, is not fully understood. By utilizing comparative acetylome and transcriptome analyses, we identified a set of histone and nonhistone proteins as potential substrates of Gcn5-mediated acetylation in *M. oryzae*. By genetic and biochemistry analyses, we revealed the cell biology and biological function of some of these substrates. Our results reveal the roles of Gcn5 in epigenetically regulating gene transcription as a histone modifier and in executing broad regulation of multiple proteins and sites across several key metabolism and stress response pathways involved in nutrient signaling and energy homeostasis. Proteins involved in regulating carbon metabolism were particularly targeted by Gcn5, which indicates the potential for regulation during changes in nutrient availability. Our large-scale inventory of acetylated proteins and sites in the *M. oryzae* mycelia significantly expands our understanding of how Gcn5 may regulate fungal pathogenic differentiation via epigenetic regulation of global gene expression as well as posttranslational modifications (PTM) of nonhistone proteins. We summarized the metabolic pathways potentially subject to Gcn5 regulation via acetylation in Fig. 8. Multiple substrates of Gcn5 were identified distributing across numerous cellular compartments and carrying a broad range of cellular functions, such as epigenetic regulation of gene expression via histone acetylation in nuclei, regulating carbohydrate metabolism (glycolysis and PPP), fatty acid metabolism, autophagy, and cell wall β-glucan biosynthesis in the cytosol, regulating oxidative phosphorylation and TCA cycle in mitochondria, and regulating protein synthesis in ribosomes. This may at least partially explain the pleiotropic effects observed in the *gcn5*Δ (KO) mutant. In this study, we verified that Pk is subjected to Gcn5 acetylation, and likely on the lysine 188 residue critical for its degradation in an autophagy-dependent manner. Regulation of Pk turnover via acetylation and autophagic degradation contributes to proper conidiation and host infection, likely through fine-tuning glycerol production through glycolysis to suit different physiological/environmental conditions. Further verification of more Gcn5 substrates and the functional role of acetylation on such a substrate(s) during phototropic induction of asexual differentiation and/or host infection will broaden our understanding of fungal pathogenicity and the corresponding regulatory mechanisms. Future studies will also be needed to address the stoichiometry of acetylation at different sites and possible competition between different acyl modifications.

## MATERIALS AND METHODS

### Growth conditions and strains used in this study.

Magnaporthe oryzae wild-type strain B157 (field isolate, mat1-2) was obtained from the Fungal Patho-Biology Group (Temasek Life Sciences Laboratory, Singapore). The *GCN5OX* strain was generated and verified as described previously (21). *M. oryzae* strains were cultivated on solid prune agar (PA) medium (39) at 28°C for 6 days, followed by 12-h light exposure.

### LC-MS/MS analysis.

Total protein lysates (10 mg) from WT and OX strain (with three independent biological replications) were digested with trypsin for 20 h at 37°C, followed by enrichment of acetylated peptides with the PTMScan Acetyl-Lysine Motif (Ac-K) kit (Cell Signaling Technology, catalog no. 13416S). The peptides were separated on Easy-nLC1000 liquid chromatograph (Thermo Fisher Scientific, US) with a trap column (Thermo Fisher Scientific Acclaim PepMap100; 100 μm by 2 cm; nanoViper C_18_) and analysis column (Thermo Fisher Scientific EASY column; 10 cm; ID 75 μm; 3 μm; C_18_-A2). The peptides were analyzed in a Q Exactive mass spectrometer (Thermo Fisher Scientific, US), eluted from the column with a linear solvent gradient (buffer A is 0.1% formic acid [FA]; buffer B is 84% ACN/0.1% FA) for 120 min at a flow rate of 300 nl/min (from 0 to 110 min, f gradient from 0 to 55% buffer B; from 110 to 115 min, gradient from 55 to100% buffer B; from 115 to 120 min, 100% buffer B). This LC gradient was used for all mobile phase compositions. The mass spectrometer was operated in the positive ion mode at 2.1 kV, and the capillary temperature was set at 320°C. The full scan was performed in the enhanced mode, 300 to 1,800 *m/z*.

### Database search and bioinformatic analyses.

Raw LC-MS/MS data files were processed with Maxquant software (version 1.3.0.5) for database searching, using uniprot_Magnaporthe_oryzae_39372_20160315.fasta and with the following parameters: Main search ppm, 6 ppm; Missed cleavage, 2; MS/MS tolerance ppm, 20 ppm; De-Isotopic, True; enzyme, trypsin; Fixed modification, Carbamidomethyl(C); Variable modification, Oxidation(M), Acetyl (Protein N-term), Acetyl(K); Decoy database pattern, Reverse; iBAQ, True; Match between runs, 2 min. The criteria for inclusion of protein or peptide were set as follows: a peptide FDR of ≤0.01 or a protein FDR of ≤0.01.

The comparative proteomics strategy was conducted by employing data-dependent nano-LC–MS/MS analysis (40), using Perseus (version 1.3.0.4) (41). Quantitation of peptides was performed on the processed data using Perseus, which uses MS intensities from raw LC-MS data to find statistical proteomic differences between two samples. Prior to ratio quantitation in Perseus, peak intensities from all six LC-MS/MS runs were normalized by the total ion chromatogram intensity. Student’s *t* test was performed for statistical analysis, and statistical filters were set with a *P* value of ≤0.05 to detect differential protein ratios between two samples. Statistical analysis of the AcK motif was performed with motif-x (38) and a well-established methodology (42).

### Functional annotation and protein interaction network of acetylated proteins.

Annotation of identified proteins from the Perseus processing was performed with Blast2go suite (43) under a trial license, and Interpro scan (44) (http://www.ebi.ac.uk/InterProScan/). Analysis of Gene Ontology (GO) terms and KEGG pathway enrichment of differentially acetylated proteins was performed by Applied Protein Technology Co. Ltd. (Shanghai, China).

### Epifluorescence microscopy.

*M. oryzae* cells expressing fluorescent protein-fused chimera were grown under requisite conditions. Epifluorescence microscopy was performed using Observer Z1 and a sCMOS camera (PCO Edge, Germany).

### Immunoblotting and immunoprecipitation.

Total protein extraction follows the established protocol (21). Total protein concentration was measured using the Bio-Rad Protein Assay (catalog no. 500-0006). Samples were resolved by 12% SDS-PAGE, followed by Western blotting with anti-RFP (rabbit; 1:1,000; Clontech, catalog no. R10367), or anti-GFP (rabbit; 1:5,000; Invitrogen Molecular Probes, catalog no. A6455). The secondary antibody was anti-rabbit (1:20,000; HiSecTMHRP-conjugated, Ab202) followed by detection using the SuperSignal West Pico chemiluminescent substrate (Pierce, catalog no. 34080). Total lysates from the GFP-Gcn strain were extracted with the lysis buffer (10 mM Tris-HCl [pH 7.5], 150 mM NaCl, 0.5 mM EDTA, 0.5% Nonidet P-40 substitute [Sigma-Aldrich, IGEPAL CA-630, I3021], with 2 mM PMSF and proteinase inhibitor cocktail [Sigma-Aldrich, cOmplete, catalog no. 11836170001]). Such nondenatured lysates were subject to immunoprecipitation with GFP-Trap (Chromo Tek, gta-20). The IP proteins (about 30 μg) were digested in solution using trypsin (Sigma-Aldrich, catalog no. T7409) for 20 h at 37°C, and the digested peptides were subjected to Nano-HPLC/ESI-ion trap-MS/MS analysis with a Q Exactive mass spectrometer (Thermo Fisher Scientific, US). Raw MS data files were processed and analyzed using Mascot2.2 (Matrix Science, UK) for database search with the following parameters: Database, UniProt; Taxonomy, Magnaporthe oryzae (39372); Enzyme, trypsin; Dynamical modifications, Oxidation (M); Fixed modifications, Carbamidomethyl (C); Max Missed Cleavages, 2; ProteomicsTools, 3.1.6; Filter by score, ≥20.

### RNA-seq and transcriptome analyses.

RNeasy mini kit (Qiagen, catalog no. 74104) was used for total RNA extraction from WT and OX strains. Next-generation sequencing library preparations were constructed according to the manufacturer’s protocol (NEBNext Ultra RNA Library Prep kit for Illumina). The poly(A) mRNA isolation was performed using the NEBNext Poly(A) mRNA Magnetic Isolation Module (New England BioLabs [NEB]). mRNA fragmentation and priming were performed using NEBNext First Strand Synthesis Reaction Buffer and NEBNext Random Primers. First-strand cDNA was synthesized using ProtoScript II Reverse Transcriptase and the second-strand cDNA was synthesized using Second Strand Synthesis Enzyme Mix. The purified double-stranded cDNA（by AxyPrep Mag PCR Clean-up [Axygen]) was then treated with End Prep Enzyme Mix to repair both ends and add a dA tail in one reaction, followed by a T-A ligation to add adaptors to both ends. Size selection of adaptor-ligated DNA was then performed using AxyPrep Mag PCR Clean-up (Axygen), and fragments of ∼360 bp (with the approximate insert size of 300 bp) were recovered. Each sample was then amplified by PCR for 11 cycles using Illumina P5 and P7 adaptor primers, both carrying sequences which can anneal with the flow cell to perform bridge PCR and P7 primer carrying a six-base index allowing for multiplexing. The PCR products were cleaned up using AxyPrep Mag PCR Clean-up (Axygen), validated using an Agilent 2100 bioanalyzer (Agilent Technologies, Palo Alto, CA, USA), and quantified with a Qubit 2.0 fluorometer (Invitrogen, Carlsbad, CA, USA). Then libraries with different indices were multiplexed and loaded on an Illumina HiSeq instrument according to the manufacturer’s instructions (Illumina, San Diego, CA, USA). Sequencing was carried out using a 2 × 150-bp paired-end (PE) configuration; image analysis and base calling were conducted by the HiSeq Control Software (HCS) plus OLB plus GAPipeline-1.6 (Illumina) on the HiSeq instrument.

In order to remove technical sequences, including adaptors, PCR primers, or fragments thereof, and quality of bases lower than 20, pass filter data of fastq format were processed by Trimmomatic (v0.30) to be high-quality clean data. Then, the clean data were aligned to the reference genome via software Hisat2 (v2.0.1).

Genes with a fold change of ≥2 and a false discovery rate (FDR) of <0.05 in a comparison of significant differentially expressed genes (DEGs) were subjected to enrichment analysis of GO functions and KEGG pathways. GO-TermFinder was used identifying Gene Ontology (GO) terms that annotate a list of enriched genes with a significant *P* value of less than 0.05. The formula used to calculate the *P* value was:

$$P=1-\overset{n\;-\;1}{\underset{i\;=\;0}{\sum }}\frac{\left(\overset{M}{\underset{i}{}}\right)\left(\overset{N\;-\;M}{\underset{n\;-\;i}{}}\right)}{\left(\overset{N}{\underset{n}{}}\right)}$$

where *N* is the number of all genes with GO annotation, *n* is the number of DEGs in *N*, *M* is the number of all genes annotated to specific pathways, and *m* is the number of DEGs in *M*. The calculated *P* value went through FDR Correction with a FDR of ≤0.05 as the threshold.

KEGG (Kyoto Encyclopedia of Genes and Genomes) is a collection of databases dealing with genomes, biological pathways, diseases, drugs, and chemical substances (http://en.wikipedia.org/wiki/KEGG) (45). Enrich significant differential expression gene in KEGG pathways was performed with in-house script of the Genewiz company. The calculating formula is the same as that in GO analysis.

### Reverse transcriptase PCR.

Reverse transcriptase PCR (RT-PCR) was performed using a one-step RT-PCR kit (Qiagen, catalog no. 210212). The primers for RT-PCR are shown in Table S1 in the supplemental material.
