# Supplementary figures and images for: Label-Free Quantitative Proteomics of Lysine Acetylome Identifies Substrates of Gcn5 in Magnaporthe oryzae Autophagy and Epigenetic Regulation
Source: mSystems. 2018 Nov 20;3(6):e00270-18. doi: 10.1128/mSystems.00270-18 (PMC6247014; doi:10.1128/mSystems.00270-18)

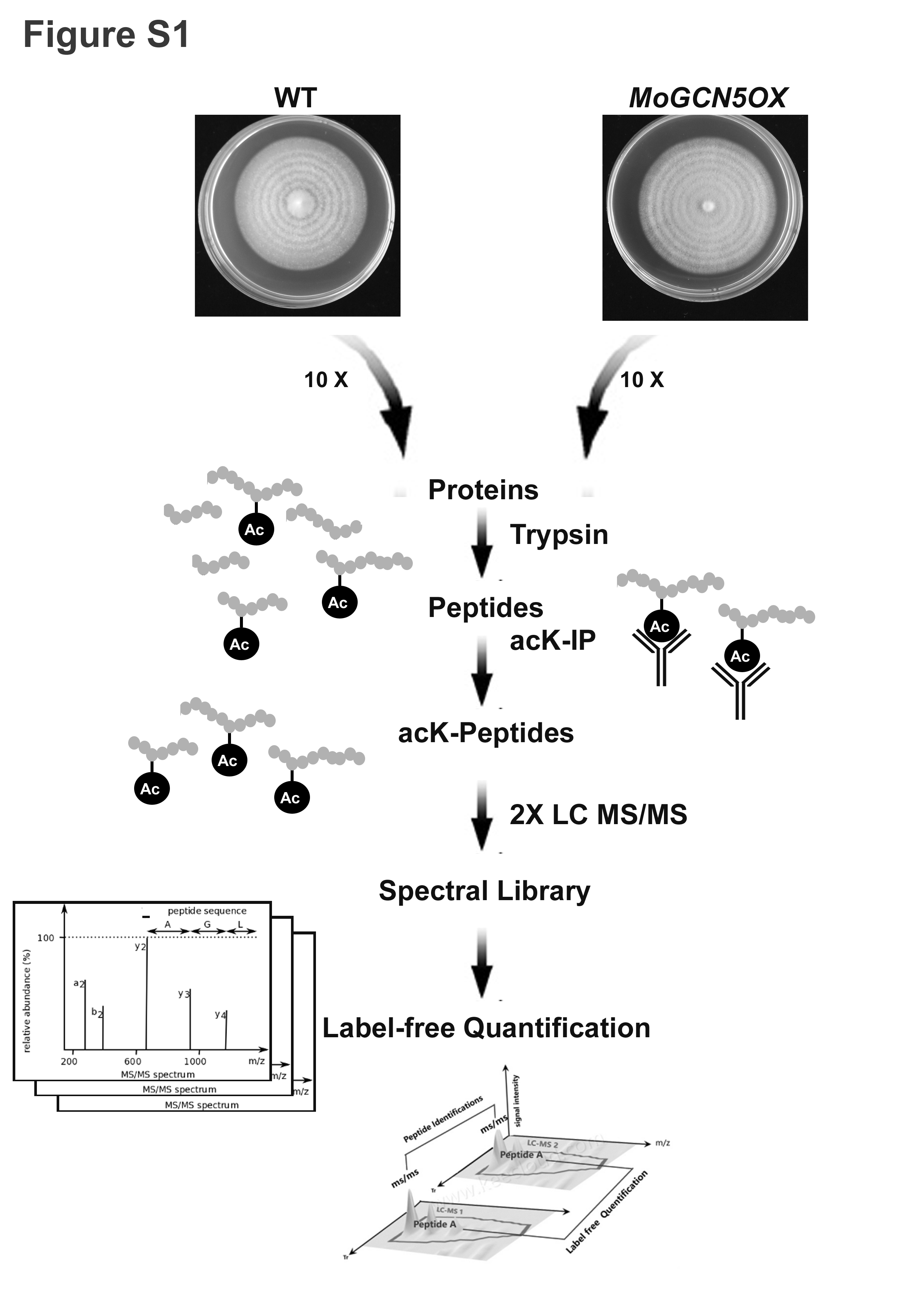

Supplement: FIG S1 [file sys006182295sf1.tif]

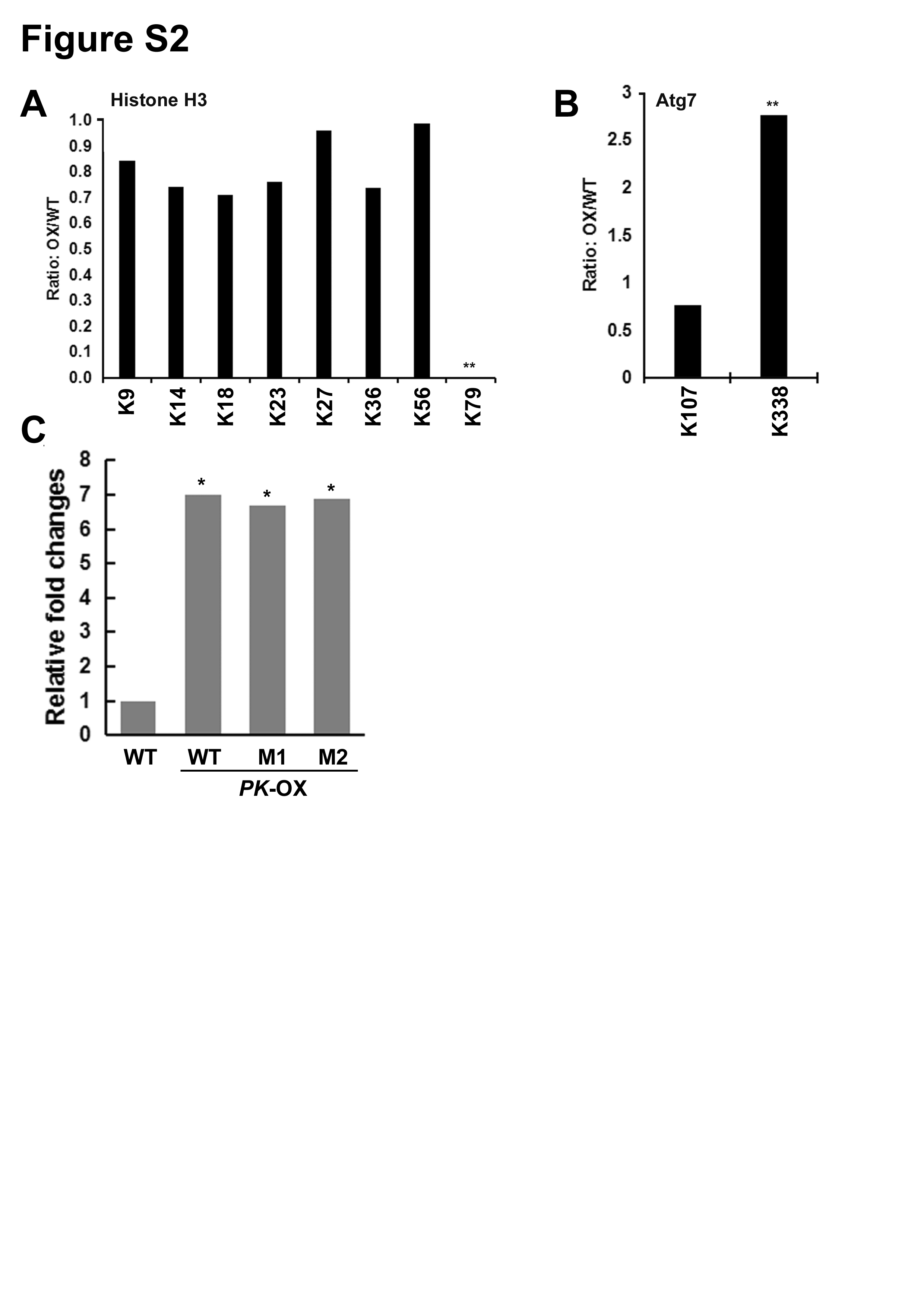

Supplement: FIG S2 [file sys006182295sf2.tif]
