# Supplementary material for: Label-Free Quantitative Proteomics of Lysine Acetylome Identifies Substrates of Gcn5 in Magnaporthe oryzae Autophagy and Epigenetic Regulation
Source: mSystems. 2018 Nov 20;3(6):e00270-18. doi: 10.1128/mSystems.00270-18 (PMC6247014; doi:10.1128/mSystems.00270-18)
Supplement: TABLE S1 [file sys006182295st1.docx]

**Table S1** Selected Differentially Expressed Genes for verification by RT-PCR.

|  |  | **RNAseq Results** | | **Primers for RT PCR verification** | |
| --- | --- | --- | --- | --- | --- |
| **Gene function** | **Gene Number** | **fold change** | **p value** | **RT-F** | **RT-R** |
| **Autophagy genes** |  |  |  |  |  |
| ATG5 | MGG_09262 | 0.967442785 | 0.911902882 | gacaccttcctcaacggcgtcaag | tggcacgggagcgccgtgaagcacc |
| ATG7 | MGG_07297 | 0.993591397 | 0.98227265 | GGCCTCTACCAGCCAATGTCGGAGAG | CGCGGCAGACCCCTTCGGAAGGGTCC |
| ATG14 | MGG_03698 | 0.652229151 | -0.616549173 | gggccgactgggatctcgtccacga | acatggcaggatcctccttggcgag |
| **Pigmentation** |  |  |  |  |  |
| Pigment | MGG_07219 | 3.134379149 | 0.0000236 | gaggatgtcgacaacatcctgggcc | ccggccataccgacaatggccagcttg |
| Pigment | MGG_05059 | 3.160897854 | 0.000183975 | GAGCGATGAGATAACCTTCTCAG | CTCGCCCCAGCGGATGTCGGGC |
| **Possible light sensor** |  |  |  |  |  |
| Bacteriodhodopsin-like | MGG_09015 | 6.668700766 | 1.3E-10 | ccggggcggccatcaagaagccgct | ctactgcagaggcgtatttgcggc |
| **Oxidative stress related** |  |  |  |  |  |
| FAD-binding | MGG_01941 | 3.645666859 | 0.00000443 | CGCCCGCCGGTTTGGTGACACTCC | GCGCGACCGCCAAGGACCATGGTCTCGT |
| FAD-binding | MGG_10760 | 17.7021533 | 2.83E-08 | gggggaaacagacaaaaggcccatc | gtagcttgtcctgggatctgacccagc |
| Stress relatedTF | MGG_09273 | 3.18149299 | 0.000166037 | GCCTCGCAAGCCGATCAAGAAGC | GTCGAGGGTCGGGGCCACGGGCC |
| **Signaling** |  |  |  |  |  |
| Kinase | MGG_11636 | 0.341357747 | 0.0000749 | GCAGCTGGTGGGAAGCAAATTG | ATTGCGGTTCGCGAATATGAGC |
| **Cell death** |  |  |  |  |  |
| Heterokaryon incompatibility | MGG_09107 | 0.276602727 | 0.0000033 | atgggcctcagtgagcttggggac | cctcaggcgatctgctccacgccatc |
| Cell death | MGG_00203 | 0.386416153 | 0.000389492 | GCCGTGGCCGAGATCGCCTTTCGC | CTGACGTCGTCATCCTGATCGAGC |
| NACHT domain-containing | MGG_09355 | 0.108253498 | 0.000000663 | gacctcgtgaccgtggttgccagc | ggggacaaacagagtgttgtaagtga |
| BRCT domain-containing | MGG_01032 | 0.394986208 | 0.000579031 | GGCAAGCTCGCGGTCCATGAGGCG | CGCCTGCCTTGAGAATTTCAGG |
| **Conidiation** |  |  |  |  |  |
| Conidiation-related TF | MGG_13350 | 0 | 1.44038E-21 | GCCTCGGCTGCCGGATCCGCAGAG | CGGGTACTCGAGCCCGACCTGCGG |
| Conidiation-related TF | MGG_13360 | 0 | 2.25427E-05 | actactgcatcctgcaccagggttcgg | ccgggtcaaaggtgagcgaaggcag |
