# Supplementary material for: Label-Free Quantitative Proteomics of Lysine Acetylome Identifies Substrates of Gcn5 in Magnaporthe oryzae Autophagy and Epigenetic Regulation
Source: mSystems. 2018 Nov 20;3(6):e00270-18. doi: 10.1128/mSystems.00270-18 (PMC6247014; doi:10.1128/mSystems.00270-18)
Supplement: TABLE S2 [file sys006182295st2.docx]

**Table S2** Statistical analysis of acK motif significantly increased in the OX strain.

| **#** | **Motif Logo** | **Motif** | **Motif Score** | **Foreground Matches** | **Foreground Size** | **Background Matches** | **Background Size** | **Fold Increase** |
| --- | --- | --- | --- | --- | --- | --- | --- | --- |
| 1 | [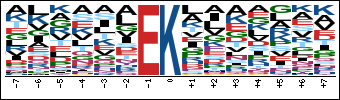](http://motif-x.med.harvard.edu/cgi-bin/jobres.pl?jobid=20161018-25279-57886684#......EK.......) | **[......EK.......](http://motif-x.med.harvard.edu/cgi-bin/jobres.pl?jobid=20161018-25279-57886684#......EK.......)** | 16.00 | 226 | 1512 | 5420 | 72479 | 2.00 |
| 2 | [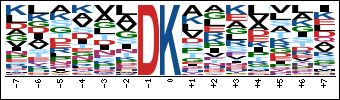](http://motif-x.med.harvard.edu/cgi-bin/jobres.pl?jobid=20161018-25279-57886684#......DK.......) | **[......DK.......](http://motif-x.med.harvard.edu/cgi-bin/jobres.pl?jobid=20161018-25279-57886684#......DK.......)** | 16.00 | 191 | 1286 | 4212 | 67059 | 2.36 |
| 3 | [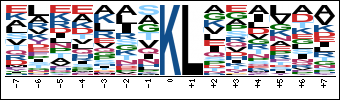](http://motif-x.med.harvard.edu/cgi-bin/jobres.pl?jobid=20161018-25279-57886684#.......KL......) | **[.......KL......](http://motif-x.med.harvard.edu/cgi-bin/jobres.pl?jobid=20161018-25279-57886684#.......KL......)** | 11.22 | 157 | 1095 | 5127 | 62847 | 1.76 |
| 4 | [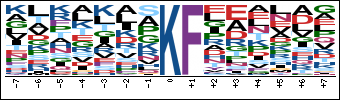](http://motif-x.med.harvard.edu/cgi-bin/jobres.pl?jobid=20161018-25279-57886684#.......KF......) | **[.......KF......](http://motif-x.med.harvard.edu/cgi-bin/jobres.pl?jobid=20161018-25279-57886684#.......KF......)** | 11.48 | 75 | 938 | 1886 | 57720 | 2.45 |
| 5 | [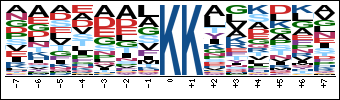](http://motif-x.med.harvard.edu/cgi-bin/jobres.pl?jobid=20161018-25279-57886684#.......KK......) | **[.......KK......](http://motif-x.med.harvard.edu/cgi-bin/jobres.pl?jobid=20161018-25279-57886684#.......KK......)** | 9.30 | 126 | 863 | 4620 | 55834 | 1.76 |
| 6 | [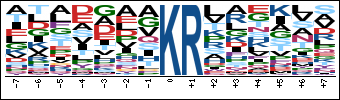](http://motif-x.med.harvard.edu/cgi-bin/jobres.pl?jobid=20161018-25279-57886684#.......KR......) | **[.......KR......](http://motif-x.med.harvard.edu/cgi-bin/jobres.pl?jobid=20161018-25279-57886684#.......KR......)** | 10.60 | 104 | 737 | 3630 | 51214 | 1.99 |
| 7 | [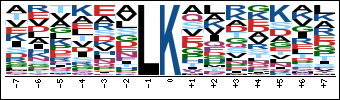](http://motif-x.med.harvard.edu/cgi-bin/jobres.pl?jobid=20161018-25279-57886684#......LK.......) | **[......LK.......](http://motif-x.med.harvard.edu/cgi-bin/jobres.pl?jobid=20161018-25279-57886684#......LK.......)** | 9.27 | 112 | 633 | 4631 | 47584 | 1.82 |
| 8 | [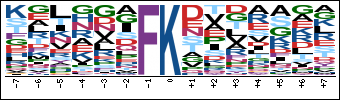](http://motif-x.med.harvard.edu/cgi-bin/jobres.pl?jobid=20161018-25279-57886684#......FK.......) | **[......FK.......](http://motif-x.med.harvard.edu/cgi-bin/jobres.pl?jobid=20161018-25279-57886684#......FK.......)** | 9.68 | 55 | 521 | 1745 | 42953 | 2.60 |
| 9 | [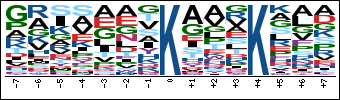](http://motif-x.med.harvard.edu/cgi-bin/jobres.pl?jobid=20161018-25279-57886684#.......K...K...) | **[.......K...K...](http://motif-x.med.harvard.edu/cgi-bin/jobres.pl?jobid=20161018-25279-57886684#.......K...K...)** | 5.78 | 62 | 466 | 2922 | 41208 | 1.88 |
